# Lipidomic characterisation discovery for coronary heart disease diagnosis based on high-throughput ultra-performance liquid chromatography and mass spectrometry†

**DOI:** 10.1039/c7ra09353e

**Published:** 2018-01-02

**Authors:** Chang Liu, Wen-jing Zong, Ai-hua Zhang, Hua-min Zhang, Yi-han Luan, Hui Sun, Hong-xin Cao, Xi-jun Wang

**Affiliations:** Sino-America Chinmedomics Technology Collaboration Center, National TCM Key Laboratory of Serum Pharmacochemistry, Laboratory of Metabolomics, Department of Pharmaceutical Analysis, Heilongjiang University of Chinese Medicine Heping Road 24 Harbin China xijunwangls@126.com +86-451-82110818 +86-451-82110818; China Academy of Chinese Medical Science Southern Street of Dongzhimen No. 16 Beijing 100700 China

## Abstract

Although many diagnostic tools have been developed for coronary heart disease (CHD), its diagnosis is still challenging. Lipids play an important role in diseases and a lipidomics approach could offer a platform to clarify the pathogenesis and pathologic changes of this disease. To the best of our knowledge, no lipidomics studies on serum have been attempted to improve the diagnosis and identify the potential biomarkers of CHD. The aim of this study was to investigate the distinctive lipid changes in serum samples of CHD patients and to identify candidate biomarkers for the reliable diagnosis of CHD using this platform. In this study, the serum lipid profiles of CHD patients were measured *via* ultra-performance liquid chromatography-G2-Si-high definition mass spectrometry combined with multivariate data analysis. A MetaboAnalyst tool was used for the analysis of the receiver operating-characteristic, while the IPA software was applied for the pathway analysis. The obtained results inferred that 33 lipid molecular species involving 6 fatty acids, 21 glycerophospholipids and 6 sphingolipids have significant differences in the serum of CHD patients. Simultaneously, 4 upstream regulatory proteins related to lipid metabolism disorders of CHD were predicted. Ten lipids have high clinical diagnostic significance according to the receiver operating-characteristic curves. This research shows that the in-depth study of lipids in the serum contributes to the clinical diagnosis of CHD and interprets the occurrence and development of CHD.

## Abstract 2

Although many diagnostic tools have been developed for coronary heart disease (CHD), its diagnosis is still challenging.

## Graphical abstract

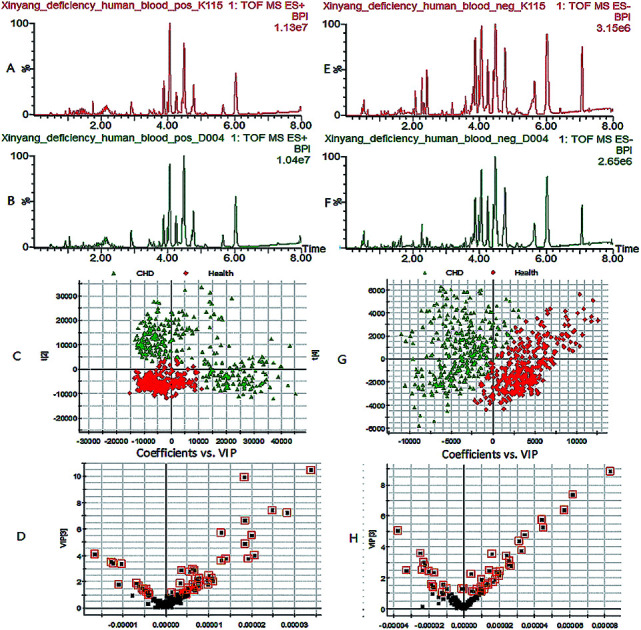

## Introduction

Coronary heart disease (CHD) is the most common cardiovascular disease, which causes severe damage to human health.^1^ Owing to lipid metabolism disorders, lipids in the artery intima accumulate, resulting in arteriosclerosis and angina in the arterial lumen and heart ischemia, thus CHD is also named ischemic heart disease (IHD).^2,3^ About 99% of CHD patients suffer from coronary atherosclerosis caused by lipid metabolism disorder, which finally leads to cardiac and hemodynamic dysfunctions.^4^ Therefore, lipid metabolism disorder is essential for CHD, and the early detection of lipids contributes to the prevention and therapy of CHD.

Lipidomics is a branch of metabolomics, which was first proposed by Han *et al.* in 2003.^5^ The goal of lipidomics is to describe lipid profiles by the identification and determination of all lipid components within a biological sample.^6^ High-throughput analytical techniques involving ultra-performance liquid chromatography, matrix-assisted laser desorption ionization time-of-flight mass spectrometry or even shot-gun methods have been used as a platform to describe individual lipids or lipid profiles. Among these methods, the main technology is electrospray ionization mass spectrometry, which exhibits high throughput, efficiency and sensitivity.^7^

Lipidomics has been applied in a series of studies on several diseases, including infectious diseases,^8,9^ cancers,^10^ neurological disorders,^11^ metabolic diseases,^12^ and cardiovascular disorders.^13^ Research shows that sphingolipids, cholesteryl esters, glycerolipids and phospholipids (including lyso- and ether-species) have potential as biomarkers for type 2 diabetes mellitus.^14^ Ganna *et al.* identified 4 lipids with potential for clinical utility, which have a causal role in CHD development. The four metabolites were lysophosphatidylcholines (18:1 and 18:2), monoglyceride (18:2) and sphingomyelin (28:1), which are regarded as risk factors for CHD.^15^ In another study, 39 endogenous metabolites were detected by proton-NMR including unsaturated fatty acid, lactic acid, alanine, glutamate, glucose, lipid, low density lipoprotein/very low density lipoprotein, betaine, phosphocholine, taurine, choline, phosphatidyl choline, and high density lipoprotein, which showed that these metabolites can serve as metabolic biomarkers of CHD patients.^16^

Therefore, systematic lipidomics analysis of the serum in CHD patients is necessary. In this study, we applied ultra-performance liquid chromatography-G2-Si-high definition mass spectrometry (UPLC-G2-Si-HDMS) analysis combined with multivariate data analysis for the lipids profiles and pathway analysis of serum in CHD patients. A receiver operating-characteristic curve (ROC) was utilized for the analysis of lipids with clinical diagnostic value, and the prediction of upstream proteins was also performed by IPA.

## Experimental methods

### Subjects

Healthy people (*n* = 445) and CHD patients (*n* = 375) at the age of 35 to 80 were studied as the control group and CHD group, respectively. All cases were from 5 hospitals including First Affiliated Hospital of Heilongjiang University of Chinese Medicine, The Clinic of Chinese Academy of Chinese Medical Sciences, Dongzhimen Hospital Affiliated with Beijing University of Chinese Medicine, Shijiazhuang Traditional Chinese Medicine Hospital and Zhengzhou Traditional Chinese Medicine Hospital. All participants signed informed consent with no intake of cigarettes, food and drinks with caffeine and preservatives for a week before the study. CHD patients were diagnosed by coronary angiography examination with diagnostic criteria from the “Treatment Guide of Stable Angina” and “Diagnosis and Treatment Recommendations of Unstable Angina”. Patients suffering from psychosis, infectious diseases, systemic diseases, cardiopulmonary insufficiency and pregnant or lactating women were excluded from this study. All experiments were performed in compliance with the Declaration of Helsinki and approved by the ethics committee at Heilongjiang University of Chinese Medicine. Informed consents were obtained from the human participants in this study.

### Samples processing

10 mL venous blood of all participants was collected and centrifuged at 13 000 rpm for 10 min at 4 °C to separate the upper serum. Then, 800 μL methanol was added to 200 μL upper serum to precipitate the proteins in each sample. After mixing *via* eddy current oscillation, samples were centrifuged again and then dried under nitrogen gas at 45 °C. The residue was removed using 200 μL methanol. Before the lipidomics analysis, samples were centrifuged again and filtered using a 0.22 μM membrane.

### Lipids analysis conditions

Quality control (QC) samples containing all the information of each group were used optimize the conditions of UPLC-G2-Si-HDMS. In the entire process of data collection, the QC samples were first analyzed and inserted into the sequence on a regular basis to ensure the stability, repeatability and reproducibility of the instrument.

The separation of lipid profiles was performed on an Acquity UPLC System (Waters, USA). After optimizing the analysis conditions, an Acquity UPLC BEH C18 column (2.1 mM × 100 mM, 1.7 μM, Waters, USA) at 50 °C and mobile phase A (HCOOH : CH_3_CN = 0.1 : 100, v/v) and B (HCOOH : H_2_O = 0.1 : 100, v/v) with a flow rate of 0.5 mL min^−1^ were chosen to obtain chromatograms with the gradients of 0–0.5 min, 5–20% A; 0.5–2.5 min, 20–60% A; 2.5–4 min, 60–66% A; 4–5 min, 66% A; 5–7.5 min, 66–86% A; and 7.5–8 min, 86–99% A.

The raw data acquisition of lipids profiles was performed on a Synapt G2-Si-HDMS (Waters, USA). The final analysis conditions were as follows: capillary voltage of 3 kV in positive ion mode and 2.5 kV in negative ion mode, cone voltage of 20 V, desolvation gas flow of 600 L h^−1^, desolvation temperature of 350 °C and source temperature of 110 °C in both ion modes. Leucine-enkephalin was chosen as a reference to calibrate the accurate mass in the centroid mode with a scan range of *m*/*z* 50 to 1000 Da. Under the conditions of 30–50 eV high-energy detection and 10–30 eV low energy detection, the MS/MS fragments were acquired for the characterization of lipids in serum samples.

### Data analysis and lipid characterization

The Progenesis QI software (Waters, USA) was used to convert all raw data for multivariate data analysis with the analysis process involving peak deconvolution and alignment, which gave the detected peaks' retention time, accurate mass and relative intensities. Unsupervised principal component analysis (PCA) and orthogonal projection to latent structures discriminate analysis (OPLS-DA) were performed using the EZinfo software (Waters, USA) to reflect the information of metabolic difference and screen the ions with a VIP value more than 1 since potential ions have a great contribution to the differences between the CHD group and the control group. The potential ions were filtered through statistical analysis with a *p* value less than 0.05. Further characterization was carried out using the Mass Fragment software with MS/MS data and exact molecular mass. The entire process of lipid characterization and annotation was based on LIPID MAPS (LIPID Metabolites and Pathways Strategy) and HMDB (Human Metabolome Databases). The IPA software was used for the prediction of related upstream regulatory proteins.

### ROC analysis

The receiver operating-characteristic (ROC) analysis was performed using the MetaboAnalyst 3.0 software and the value of the area under the curve (AUC) combined with the optimal cut-off value was used to assess the false positive rate (presented in the *x*-axis as specificity) and true positive rate (presented in the *y*-axis as sensitivity) of lipids in serum for the clinical diagnosis of CHD. In general, the closer the AUC value to 1, the better the diagnostic significance. However, if the AUC value is less than 0.5, the lipid could not be utilized for diagnosis.

## Results and discussion

### Lipid profiles analysis

**Fig. 1:**
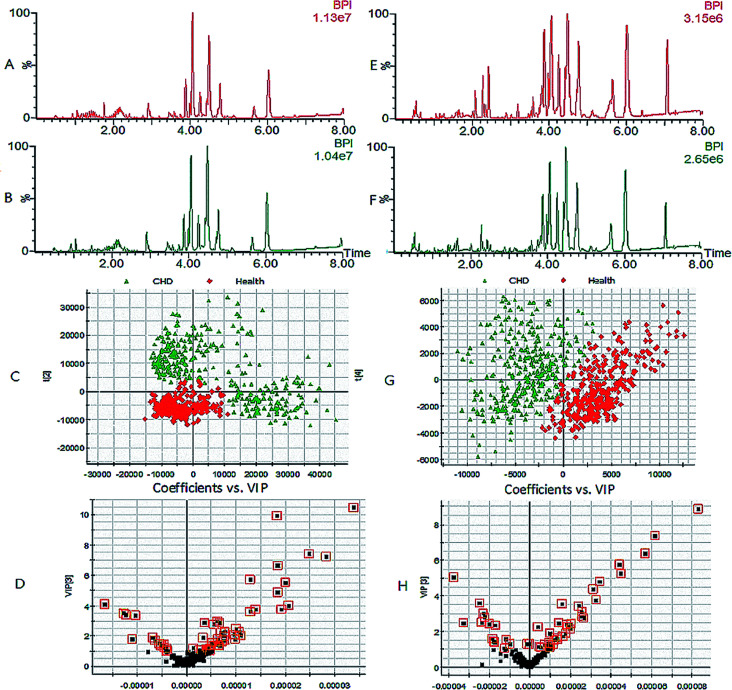
Lipid profiles based on UPLC-G2-Si-HDMS and multivariate data analysis. (A, E) Lipid profiles of healthy people; (B, F) lipid profiles of CHD patients; (C, G) 2D score plot of PCA; (D, H) VIP plot of OPLS-DA. (A–D) Positive ion mode; (E–H) negative ion mode.

**Fig. 2:**
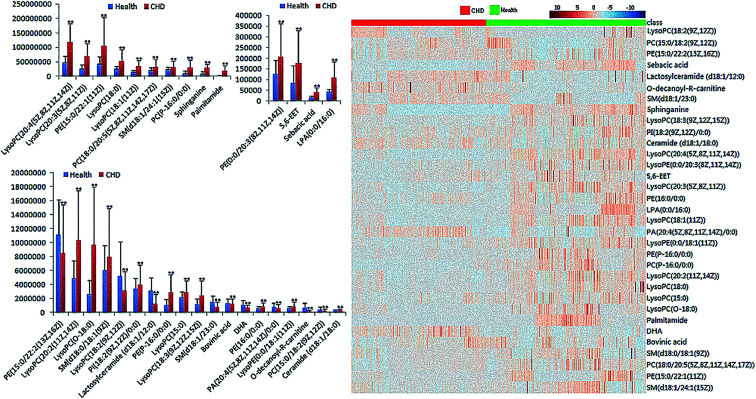
Expression of each lipid between CHD patients and healthy people and heat map of differential lipids by statistical analysis (**P < 0.05).

The best lipids profiles for the serum samples were obtained using UPLC-G2-Si-HDMS combined with the Masslynx analysis platform and the optimum analytical conditions described above. As shown in Fig. 1, the optimized analysis method of raw data and peak scanning was repeatable and stable for lipidomics research in typical base peak ion (BPI) chromatograms. Using the Progenesis QI software and EZinfo software, the differences in the lipids profiles between CHD patients and healthy people are clearly expressed in the score plot (Fig. 2). For the further detection of the exact differences, OPLS-DA analysis was applied, and two groups were well separated. In the VIP plot (Fig. 2), the differentially expressed lipids were selected based on a VIP value more than 1 and *p* value less than 0.05 after statistical analysis.

### Characterization of endogenous lipids

**Fig. 3:**
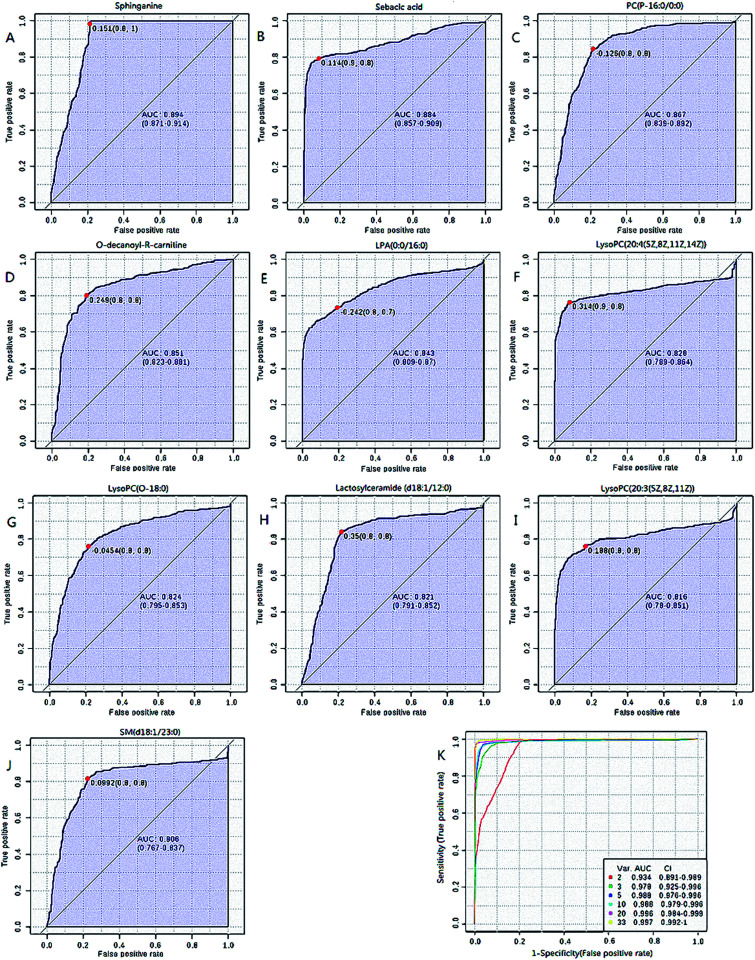
Receiver operating-characteristic (ROC) curves of lipids with top 10 AUC values (A–J) and all 33 lipid combined ROC curves (K).

Multivariate data analysis performed by the Progenesis QI and EZinfo software provided the precise molecular masses and retention times of the lipids, which have the highest sectionalization contribution. By screening the probable elemental composition of ions less than 5 ppm and obtaining MS/MS fragmentation data using the Masslynx analysis platform, 33 different lipids in CHD patients' serum samples were characterized after searching the LIPID MAPS and HMDB databases. As shown in ESI Table 1,† 33 different lipids primarily belonging to three classes were obtained, involving 21 glycerophospholipids, 6 fatty acids and 6 sphingolipids. Compared with the healthy people, the change in each serum lipid in the patients with coronary heart disease (CHD) is shown in Fig. 3. Among these lipids, 10 lipids showed a downward trend in the serum of the CHD patients, including ceramide (d18:1/18:0), SM(d18:1/24:1(15Z)), LysoPC(20:3(5Z,8Z,11Z)), LysoPC(18:1(11Z)), LysoPC(20:2(11Z,14Z)), LysoPE(0:0/18:1(11Z)), PE(P-16:0/0:0), PE(15:0/22:1(11Z)), PA(20:4(5Z,8Z,11Z,14Z)/0:0), and DHA, while the other 23 lipids showed an upward trend.

Lipid peroxidation is the primary pathogenesis of CHD, which affects the stability of plaques.^13^ Phosphatidylcholine (PC) has lytic effect on grease and promotes blood circulation, reduces the fat retention time in vessels and promotes the dissipation of atherosclerotic plaques.^17^ Lysophosphatidylcholine (LysoPC) acts as an inflammatory medium related to the proliferation and apoptosis of endothelial cells and influences the development of atherosclerosis.^18^ Phosphatidylethanolamine (PE) is the most abundant lipid in the membrane and the primary signal of cell apoptosis related to evagination of the membrane.^19^ Lysophospholipid acid (LPA) aggravates the injury and apoptosis of myocardial cells by activating the expression of NF-κB gene to increase the adhesion effect of neutrophils and monocytes.^20^ In addition, LPA causes cardiomyocyte hypertrophy and cardiac failure by facilitating the proliferation of fibroblasts and accelerating the composition of collagen in the myocardial interstitial.^21,22^ In our study, it was observed that some species of lipids such as PC(P), PE(P) and LPC(O) were altered in the blood samples of CHD patients, which are also altered in type 2 diabetes patients. According to the rank of the AUC value, LysoPC(O-18:0) and PC(P-16:0/0:0) were the eighth and ninth contributors to clinical diagnosis, respectively.

Sphingomyelin stimulated the generation of oxidizing agents, such as ROS and NO, to mediate mitochondria damage and the hypofunction of myocardial contraction.^23^ Lactosylceramide also plays an important role in the formation of atherosclerosis. Lactosylceramide makes myocardial cells hypertrophic and stimulates vascular smooth muscle proliferation by enhancing the activity of superoxide radical mediated p44MAPK and protein kinase.^14,24^ Bovinic acid inhibits the formation of foam cells by regulating PGC-1α to decrease the absorption of Ox-LDL and increase the outflow of cholestenone.^25,26^ 5,6-Epoxy-8,11,14-eicosatrienoic acid (5,6-EET) is the main product of the metabolism of the anti-inflammatory arachidonic acid and has the physiological function of blood pressure regulation and signal conductive regulation related to angiogenesis, platelet aggregation, neural hormone release and leukocytes adhesion.^27^

### Diagnosis analysis

Further, ROC analysis according to the standard method described above was performed for each lipid and the combination of 33 lipids to examine whether these lipids have the potential for clinical application. The results shown in Fig. 3 and ESI Table 2,† obtained using MetaboAnalyst 3.0, indicate that the top 10 AUC values of a single lipid were more than 0.8, which has good potential for clinical diagnosis; these single lipids were DHA, *O*-decanoyl-*R*-carnitine, sphinganine, SM(d18:1/23:0), lactosylceramide (d18:1/12:0), LysoPC(20:4(5Z,8Z,11Z,14Z)), LysoPC(20:3(5Z,8Z,11Z)), LysoPC(O-18:0), PC(P-16:0/0:0) and LPA(0:0/16:0). Although the highest AUC value of single lipid was 0.894, which is less than 0.9, the AUC value of all the lipids together was 0.997. This result indicates that the combination of all the lipids show better diagnostic significance compared to a single lipid.

By evaluating the clinical diagnostic value of the 33 lipids *via* ROC analysis, the AUC value of 10 lipids was more than 0.8, which contributed to distinguishing between CHD patients and healthy people and showed greater independent diagnosis ability for CHD. Simultaneously, the combined application of 33 lipids has the largest diagnostic value and the AUC value reached 0.997. The results indicate that lipids with a relatively low AUC value also have an existence value by ROC analysis of total lipids and the single lipid diagnostic accuracy of CHD is less than the overall lipid diagnosis.

### Molecular pathway analysis

**Fig. 4:**
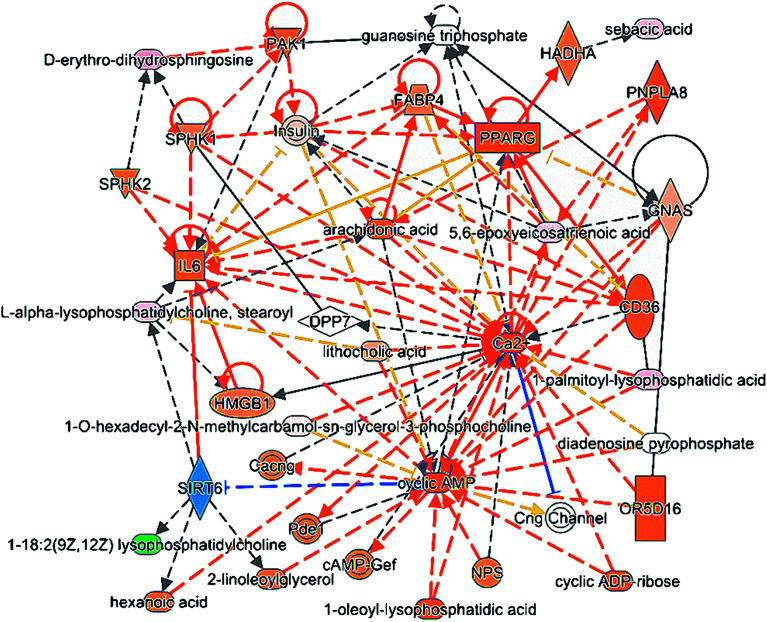
Interaction network of lipid metabolism in coronary heart disease.

Ingenuity Pathway Analysis (IPA) is an online integration analysis software based on the large life sciences database. This software is used to clarify the molecular interactions relations between chemical materials, genes, proteins and metabolites, and is particularly used as a system to predict the regulating effect of upstream proteins to lipid metabolites. Through the comprehensive analysis using the IPA software, the upstream regulatory proteins including SIRT6, HADHA, PNPLA8 and SPHK were the most related to the lipid metabolic markers for CHD and highly related to the network of cell signaling, molecular transport, vitamin and mineral metabolism and the network of developmental disorder, hereditary disorder, and lipid metabolism. The detailed interactions of all the lipids and upstream proteins are displayed in Fig. 4. The metabolites in light pink indicate high expression in the CHD group, while those in green ink indicate low expression in the CHD groups. The proteins in orange ink have predicted activation of metabolites, while those in blue ink have predicted inhibition of metabolites.

Silent information regulator 6 (SIRT6) has a close relationship to lysophosphatidylcholine based on the predictions of a large database and is the sole protein with an inhibition trend to lipid in the entire interaction network by IPA. SIRT6 has been proven to reduce foam cell formation *via* an autophagy-dependent pathway against atherosclerosis and exhibits an anti-hypertrophic effect by inhibiting NF-κB activation.^28,29^ PNPLA 8 (patatin-like phospholipase domain-containing protein 8) has a close relationship to 5,6-EET in the predicted interaction network and has a significant positive relation to the expression of transcription factors LXRα, PPARa, and SREBP2, which are all closely related with fat deposition.^30^ Hydroxyacyl-CoA dehydrogenase (HADHA) leads to dehydrogenation to the corresponding coenzyme A in mitochondria, which is the major limiting step of fatty acid β oxidation to provide abundant energy for myocardial function at the physiological state.^31^ Sphingosine kinase 1 (SPHK1) is the major rate-limiting enzyme of sphingosine 1-phosphate (S1P) and catalyzes the generation of S1P to promote cell survival and protect cells from apoptosis by regulating TNF-α and NF-κB.^32^

Lipid is the generic term for fat and lipoids and consists of fatty acid, glycerolipid, sphingolipid, pregnenolone lipids, glyceryl phosphatide, sterol lipids, and glycolipids. Lipid is a type of important biological macromolecule with a variety of biological functions and is closely related to the occurrence and development of CHD as a known risk factor. Therefore, the research of lipid metabolism disorder in CHD patients helps to better understand CHD. In this study, 33 lipids in serum related to CHD were characterized using the UPLC-G2-Si-HDMS analysis platform combined with multiple database search and the results suggest that the lipid metabolism disorder in CHD patients primarily refers to the lipid pathways of glycerophospholipid metabolism, sphingolipid metabolism and fatty acids metabolism.

Through the comprehensive analysis using the IPA software, upstream regulatory proteins including SIRT6, PNPLA8, HADHA and SPHK1 are the most related to the lipid metabolic markers for CHD. Looking for upstream regulatory proteins based on lipid markers could further clarify the mechanism and changing process of CHD. However, the specific regulatory mechanism of these proteins to lipid markers related to CHD still needs deep biological validation.

## Conclusion

In this study, serum lipids profiles of CHD patients were determined using the UPLC-G2-Si-HDMS analysis platform combined with multivariate data analysis and multi-database search. In total, 33 CHD related lipids are primarily related to glycerophospholipid metabolism, sphingolipid metabolism and fatty acids metabolism. 10 lipids have significant clinical diagnostic significance. The ROC analysis certified that the lipid profiles of CHD patients have great disorder and the characterized lipids show great diagnostic value for clinical application. The results proved that a variety of blood lipid profiles were disordered in the entire course of CHD and the research on lipids and upstream proteins could help in the better understanding and prevention of CHD. Consequently, CHD can be controlled to improve the survival rate of patients.

## Conflicts of interest

The authors declare that there is no financial interest in relation to the publication of this paper.

## Supplementary Material

RA-008-C7RA09353E-s001
