# Supplementary material for: Lipidomic characterisation discovery for coronary heart disease diagnosis based on high-throughput ultra-performance liquid chromatography and mass spectrometry
Source: RSC Adv. 2018 Jan 2;8(2):647–54. doi: 10.1039/c7ra09353e (PMC9076928; doi:10.1039/c7ra09353e)
Supplement: RA-008-C7RA09353E-s001 [file RA-008-C7RA09353E-s001.pdf]

Electronic Supplementary Material (ESI) for RSC Advances.
This journal is © The Royal Society of Chemistry 2017

Supplementary table 1 Information of the characterized lipids in serum samples of CHD patients

No	Rt (min)	M/Z	Description	Ion mode	Formula	PPM	ID
1	7.28	279.2326	Bovinic acid	M-H	C18H32O2	-1.4	LMFA01030118
2	6.92	327.2326	DHA	M-H	C22H32O2	-1.2	LMFA01030185
3	1.96	201.1125	Sebacic acid	M-H	C10H18O4	-3.8	LMFA01170006
4	4.42	319.2277	5,6-Epoxy-8,11,14-eicosatrienoic acid	M-H	C20H32O3	-0.5	LMFA03080002
5	2.64	316.2482	O-decanoyl-R-carnitine	M+H	C17H33NO4	-0.2	LMFA07070006
6	6.75	256.2656	Palmitamide	M+H	C16H33NO	3.1	LMFA08010009
7	1.86	742.5402	PC(15:0/18:2(9Z,12Z))	M-H	C41H78NO8P	1.3	LMGP01010543
8	7.93	808.5854	PC(18:0/20:5(5Z,8Z,11Z,14Z,17Z))	M+H	C46H82NO8P	0.4	LMGP01010805
9	5.96	480.3112	LysoPC(15:0)	M-H	C23H48NO7P	3.5	LMGP01050016
10	5.64	524.3733	LysoPC(18:0)	M+H	C26H54NO7P	4.2	LMGP01050026
11	1.37	520.3338	LysoPC(18:2(9Z,12Z))	M+H	C26H50NO7P	-1.4	LMGP01050035
12	3.65	518.3272	LysoPC(18:3(9Z,12Z,15Z))	M+H	C26H48NO7P	0.9	LMGP01050038
13	4.04	544.3444	LysoPC(20:4(5Z,8Z,11Z,14Z))	M+H	C28H50NO7P	3.4	LMGP01050048
14	5.11	548.3739	LysoPC(20:2(11Z,14Z))	M+H	C28H54NO7P	5.3	LMGP01050132
15	4.54	522.3593	LysoPC(18:1(11Z))	M+H	C26H52NO7P	0.4	LMGP01050138
16	4.43	546.3596	LysoPC(20:3(5Z,8Z,11Z))	M+H	C28H52NO7P	1.6	LMGP01050139
17	6.44	510.3936	LysoPC(O-18:0)	M+H	C26H56NO6P	3.6	LMGP01060014
18	4.79	480.3459	PC(P-16:0/0:0)	M+H	C24H50NO6P	2.2	LMGP01070006
19	7.93	760.5878	PE(15:0/22:1(11Z))	M+H	C42H82NO8P	3.6	LMGP02010470
20	1.89	758.5719	PE(15:0/22:2(13Z,16Z))	M+H	C42H80NO8P	3.2	LMGP02010471
21	4.43	452.2788	PE(16:0/0:0)	M-H	C21H44NO7P	1.2	LMGP02050002
22	4.74	478.2959	LysoPE(0:0/18:1(11Z))	M-H	C23H46NO7P	4.1	LMGP02050039
23	4.4	502.2951	LysoPE(0:0/20:3(8Z,11Z,14Z))	M-H	C25H46NO7P	2.4	LMGP02050050
24	4.76	436.2848	PE(P-16:0/0:0)	M-H	C21H44NO6P	3.3	LMGP02070001
25	3.86	595.2911	PI(18:2(9Z,12Z)/0:0)	M-H	C27H49O12P	3.6	LMGP06050010
26	4.69	457.2369	PA(20:4(5Z,8Z,11Z,14Z)/0:0)	M-H	C23H39O7P	1.8	LMGP10050013
27	4.48	409.2396	LPA(0:0/16:0)	M-H	C19H39O7P	3.7	LMGP10050042
28	3.39	302.3066	Sphinganine	M+H	C18H39NO2	4.2	LMSP01020001
29	3.89	564.5312	Ceramide (d18:1/18:0)	M-H	C36H71NO3	-3.8	LMSP02010006
30	7.94	813.6754	SM(d18:1/24:1(15Z))	M+H	C47H93N2O6P	-1.1	LMSP03010007
31	7.29	731.6023	SM(d18:0/18:1(9Z))	M+H	C41H83N2O6P	-3.2	LMSP03010031
32	2.87	801.6783	SM(d18:1/23:0)	M+H	C46H93N2O6P	-2.6	LMSP0301078
33	2.59	806.5641	Lactosylceramide (d18:1/12:0)	M+H	C42H79NO13	2.0	LMSP0501AB02

Supplementary table 2 ROC analysis for the lipid metabolites.

No	Description	AUROC	T-statistics	Log2 fold change
1	Sphinganine	0.894	9.30E-145	-2.3345
2	Sebacic acid	0.88	4.41E-66	-1.1697
3	PCP-160/00	0.867	3.15E-78	-1.6938
4	O-decanoyl-R-carnitine	0.852	2.87E-52	3.1174
5	LPA00/160	0.842	1.58E-79	-1.0108
6	LysoPC2045Z,8Z,11Z,14Z	0.828	9.25E-13	-0.8536
7	LysoPCO-180	0.824	8.42E-55	-1.6484
8	Lactosylceramide d181/120	0.820	9.30E-40	2.4299
9	LysoPC2035Z,8Z,11Z	0.816	3.66E-09	-0.9182
10	SMd181/230	0.806	1.41E-48	1.9188
11	LysoPC18111Z	0.797	1.96E-05	-0.6092
12	LysoPC20211Z,14Z	0.761	5.54E-06	-0.5584
13	Palmitamide	0.752	5.68E-66	-2.1253
14	PC150/1829Z,12Z	0.731	6.45E-31	1.5293
15	LysoPC180	0.730	0.7134805	-0.0569
16	PEP-160/00	0.729	8.43E-29	-0.9149
17	DHA	0.716	1.81E-24	0.6531
18	PE160/00	0.688	6.10E-03	-0.1976
19	LysoPE00/2038Z,11Z,14Z	0.688	9.10E-08	-0.4071
20	LysoPE00/18111Z	0.683	5.43E-05	-0.2634
21	LysoPC1839Z,12Z,15Z	0.669	7.87E-04	-0.4013
22	LysoPC150	0.655	2.29E-02	-0.1545
23	5,6-Epoxy-8,11,14-eicosatrienoic acid	0.648	7.43E-14	-0.9743
24	PA2045Z,8Z,11Z,14Z/00	0.639	1.65E-12	0.7070
25	LysoPC1829Z,12Z	0.625	7.87E-12	0.8179
26	PE150/22213Z,16Z	0.622	7.13E-14	0.9972
27	SMd181/24115Z	0.597	3.66E-04	-0.2439
28	Bovinic acid	0.594	1.06E-06	0.3508
29	PC180/2055Z,8Z,11Z,14Z,17Z	0.582	4.07E-02	-0.1869
30	Ceramide d181/180	0.578	6.32E-05	0.4694
31	PI1829Z,12Z/00	0.571	7.24E-04	0.4160
32	PE150/22111Z	0.522	5.14E-01	0.1041
33	SMd180/1819Z	0.500	3.03E-04	0.5250
